# The effect of endurance exercise and rosehip extract supplementation on the expression of P53 and cytochrome C genes in male rat heart

**DOI:** 10.34172/jcvtr.2022.31599

**Published:** 2022-12-31

**Authors:** Mitra Abdollahi-Diba, Jabar Bashiri, Hadi Pourmanaf, Vahid Fekri-Kourabbaslou

**Affiliations:** ^1^Department of Exercise Physiology, Faculty of Physical Education and Sports Sciences, Tabriz University, Tabriz, Iran; ^2^Department of Exercise Physiology, Faculty of Physical Education and Sports Sciences, Islamic Azad University, Tabriz Branch, Tabriz, Iran; ^3^Department of Exercise Physiology, Faculty of Physical Education and Sports Sciences, Tehran University, Tehran, Iran; ^4^Department of Exercise Physiology, Faculty of Physical Education and Sports Sciences, Kharazmi University, Tehran, Iran

**Keywords:** Aerobic Exercise, Apoptosis, Cytochrome C, Myocardium, Rosehip

## Abstract

***Introduction:*** Considering the effect of apoptosis on cardiovascular disease, this study aimed to determine the combined effect of endurance exercise and rosehip extract supplementation on the expression of P53 and cytochrome C genes in the myocardium of male rats.

***Methods:*** A total of 35 male rats were randomly divided into five groups (n=7) as follows: endurance exercise+rosehip extract supplementation (Ex+Supp), endurance exercise (Ex), rosehip extract supplementation (Supp), six-month control (Con2), and three-month control (Con). The subjects in Ex+Supp and Ex groups performed endurance exercise (running on a treadmill at 24-33 m/min for 10-60 min) for 12 weeks, five times a week. Subjects in Ex+Supp and Supp groups consumed 1000 milligrams/ kilogram of rosehip extract for 12 weeks. Also, Con and Con2 groups did not receive any intervention. To RNA extraction and synthesis cDNA and evaluate the P53 and cytochrome C genes of the myocardium of rats, RT-PCR analysis was used.

***Results:*** Neither endurance exercise nor rosehip alone nor together significantly affected the expression of cytochrome C and P53 genes in the heart muscle of male rats (*P*>0.05). Also, endurance exercise (*P*=0.001) and rosehip supplementation (*P*=0.002) alone and in interaction (*P*<0.01) had a significant effect on body weight, myocardium weight, and the ratio of myocardium weight to body weight in male rats.

***Conclusion:*** Twelve weeks of endurance exercise accompanied with rosehip extract did not significantly affect the expression of P53 and cytochrome C genes. Further studies are suggested to confirm these results.

## Introduction

 Cardiovascular disease (CVD) is the most prevalent non-communicable disease worldwide and is considered as one of the leading causes of death globally ^1^. Mitochondria produces more than 90% of the intracellular adenosine triphosphate (ATP) used by cardiomyocytes. Thus, the heart dependent on mitochondrial function for its aerobic metabolism.^2^ Numerous risk factors and pathological mechanisms contribute to the development of CVD, such as metabolic abnormalities, excessive production of reactive oxygen species (ROS), energy deficit, and deregulation of autophagy, endoplasmic reticulum (ER) stress, and activation of apoptosis. These cellular disruptions are largely the result of mitochondrial dysfunction or malfunction.^3^

 Apoptosis is a physiological and irreversible process causing permanent cell damage. It is triggered by metabolic stress and acute or chronic ischemia. In physiological conditions, it regulates the growth and proliferation of cells in the body, prevents the development of various malignancies, and causes the establishment of cellular homeostasis.^3-5^ Additionally, it has been linked to various pathophysiological conditions and diseases, including CVD, and It has become clear that apoptosis plays a role in developing atherosclerosis.^6,7^ This physiological process often occurs through two classical intracellular and extracellular pathways: caspase activation^8^ and the BCL-2 family.^9,10^ Apoptosis can be activated by increased P53 expression in response to oxidative stress in the cells. P53 is a transcriptional factor that controls the expression of several pro-apoptotic genes, such as Bax and Bid, which can cause DNA fragmentation.^11,12^

 On the other hand, cytochrome C is one of the essential components of the electron transfer chain, which activates caspases during apoptosis. The separation of cytochrome C from the electron transport chain disrupts energy production and leads to cell death.^9^ Activation of the P53 gene and increased cytochrome C release are observed in response to various cellular stress factors such as DNA damage, severe hypoxia, cellular aging, and high oxidative stresses.^13^ Therefore, researchers are looking for appropriate solutions to protect the heart against oxidative stress and possible damage. Meanwhile, using herbal antioxidant supplements and exercise has attracted the attention of researchers.

 Regular exercise is an effective program for treating and preventing CVD and reduces the CVD-linked complications.^5^ Regardless of the type of physical activity, aerobic exercise training (AET) is always advised, particularly when the goal is to enhance cardiovascular fitness. Regular AET can lower the risk for cardiovascular diseases. It seems that endurance training reduces apoptosis in skeletal and cardiac muscles through the effect on mitochondrial membrane permeability, and has also been shown to improve antioxidant capacity in patients with chronic heart failure.^5,14-16^ Therefore, it increases the expression of cell death factors, inflammation, and immunological changes in the blood.^17^ It has been shown that levels of P53 and cytochrome C proteins increase after exercise,^18^ and exercise positively regulates the P53 gene, thereby encouraging damaged cells to repair the damage, or induce self-destruction if repair is not possible.^19^

 On the other hand, rosehip (which is also called rose haw) is the pseudo fruit of the rose plant, which is rich in different types of micronutrients and phytochemicals such as phenolic compounds, tannins, flavonoids, and carotenoids.^20,21^ Studies have shown that rosehip possesses anti-obesity, anti-inflammatory, and anti-oxidative effects, and administration can reduce the risk of cardiovascular diseases.^20-22^ Thus, we hypothesized that endurance exercise accompanied by rosehip supplementation would affect the apoptotic factors P53 and cytochrome C in the heart muscle of male rats, and therefore, can improve the cardiac function and prevent CVD.

## Materials and Methods

 The present study used an animal model (three-month-old male Wistar rats 14848) in a five-group to examine the effect of three months of aerobic exercise (five days a week, grade 15% for 10-60 minutes and speed of 24-33 m/min) along with rosehip extract consumption on the expression of myocardial P53 and cytochrome C genes. All protocol designs and surgical procedures were performed in compliance with the Guide for Using Animal Subjects. The Research Ethics Committee approved the animal experimentation study at Tabriz Azad University, Iran (Ethical code: IR.IAU.TABRIZ.REC.124).

**Figure 1:**
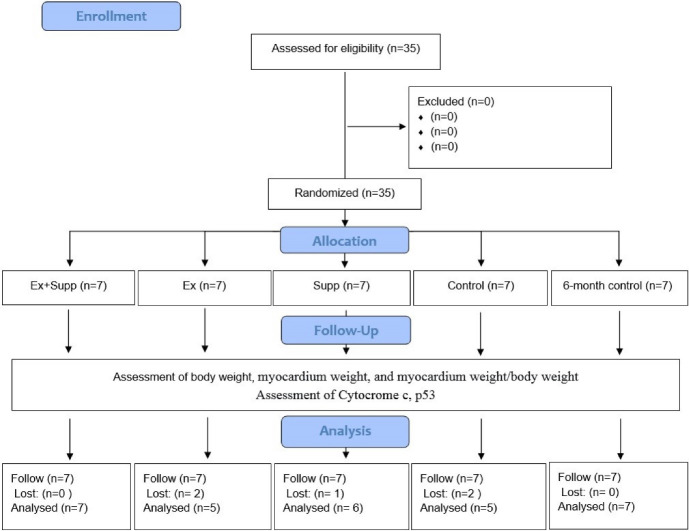
Schematic overview of study timeline (CONSORT flow diagram).

 Thirty-five two-month-old rats weighing 200 ± 20 were purchased from the Pasteur Institute, Tehran, Iran, and were housed under controlled conditions (temperature 22 ± 2°C, relative humidity 50 ± 5 %, 12:12 hours light-dark cycle)^23^ in the pet Veterinary care school of Tabriz Azad University. All subjects were fed standard chow (Pars Animal Feed Co., Tehran, Iran) and water ad libitum, and five rats were kept in each cage. Every day about 20 grams of pellets were given to each animal in each cage (two months old male rats with an average weight of 200 grams). However, with the possible increase in the weight of the animals, their daily food intake gradually increased. A silent air conditioner was used 24/7 to ventilate the airflow. After 2 weeks of acclimatization, weight-matched rats were randomly divided into five groups (n = 7), including three-month primary control (Con), six-month control (Con2), Endurance exercise (Ex), Endurance exercise + rosehip supplement (Ex + Supp), and rosehip supplement (Supp). A schematic overview of the study is presented in Figure 1.

 After familiarization with walking on a treadmill (0%, 10-15 m.min^-1^, 5-10 min.d^-1^) for one week,Ex and EX + Supp groupsexercisedfive days a week for 12 weeks on an animal treadmill (Table 1). The exercise intensity wascontrolled using the treadmill’s running speed and grade. The exercise protocol was designed based on the study of Naito et al.^23^ They determined the intensity of the exercise by estimating the maximum oxygen consumption of rats which was also matched based on the study. The grade was 15% throughout the exercise sessions. The speed also started from 24 m/min in the first week and reached 33 m/min in the twelfth week. The exercise started from 10 min/day in the first week, reached 60 min in the fifth week, and was maintained until the end. At the beginning of the exercise session, subjects ran for five min at a 10-15 m/min speed and a zero grade to warm up. Then, to achieve the desired intensity, the speed and grade of the treadmill were gradually increased in 5-10 min. At the end of this exercise program, to cool down the subjects, the grade and speed of the treadmill were reduced to zero% and 10-15 m/min, respectively. The cooling phase lasted about five minutes in the early weeks and about 10 minutes in the final weeks. The Con2 group rats were placed inside the switched-off device for 10 minutes per exercise session to simulate the treadmill’s stress level.

**Table 1:** Details of the exercising protocol used in the study

**Week**	**1**	**2**	**3**	**4**	**5**	**6**	**7**	**8**	**9**	**10**	**11**	**12**
Exercise Duration (min.d^-1^)	10	20	35	45	60	60	60	60	60	60	60	60
Treadmill grade (%)	15	15	15	15	15	15	15	15	15	15	15	15
Treadmill speed (m.min^-1^)	24	24	25	25	26	27	28	29	30	31	32	33

 Simultaneously with the Ex and Ex + Supp groups, the Supp and Ex + Supp groups received 7.5 ccs of saline solution supplemented with 1000 mg/kg rosehip extract. The measurements were performed by a sensitive digital scale (GF-300, AND, Japan), was fed to the rats by gavage after the end of each exercise protocol. During exercise protocol, no other dietary supplements were given to the animals. To extract, the dried sample of the plant was pulverized by a corrosive device, 100 grams of plant powder, and 0.96 ethyl alcohol and distilled water were added in a ratio of 1 to 1 to reach volume. After 24 hours, the solution was filtered. Then, the filtered solution was concentrated by vacuum distillation at a temperature of 50°C and a rotation speed of 70 rpm to 1.3 of the initial volume. The quality of the extract was confirmed at the Agriculture Faculty of Tabriz Islamic Azad University. Forty-eight hours after the last exercise session, all rats were anesthetized with pentobarbital sodium (50 mg·kg^-1^), and five ml of their blood was collected. Then their myocardium was removed immediately by experienced surgeons, weighed, frozen in liquid nitrogen, and stored at -70°C until further analysis.

###  RNA extraction and cDNA synthesis

 According to the kit manufacturer’s manual (Thermo K0731, USA), about 50 mg of myocardial tissue was homogenized in 1 mL of lubricating buffer before incubating at room temperature for five minutes. Then, 0.2 ml of chloroform was added to each microtube, shaken vigorously for 15 seconds, incubated for five minutes at 4°C, and then centrifuged at 13 700 g for 15 min at four°C. An equal volume of cold isopropanol was added to the isolated solution and incubated at -20 °C for 10 min. After centrifuging the microtubes and adding 1 ml of 80% ethanol, the supernatant was poured out, and the ethanol was carefully drained, allowing the alcohol to evaporate for about 20 minutes. Then 0.02 ml of water treated with diethylpyrocarbonate (DEPC) was added to each microtube. RNA concentration was measured using the UV spectrophotometry method (Eppendorf, Germany), and 260–280 portions in 1.8–2 were determined as the desired purification. cDNA synthesis was done using RevertAID TM Firs Standard kit (Fermentas., Canada) and according to the manufacturer’s instructions.

###  RT-PCR analysis

 Rotor gene-6000 (Corbett, USA) was used to measure the gene expression of these proteins. Pairs of primers for each gene were designed using Primer 3 software, synthesized by Bioneer (Bioneer, Germany), and worked with a final concentration of 100 nm. The primer sequences are presented in Table 2. For this purpose, the kit (SYBR-green Real-Time RT-PCR, TAKARA, Japan) was used. According to the kit instructions, a 20 μl amplification reaction was performed, consisting of 10 μl of the original solution (Master Mix), 0.3 μl of the Forward primer, 0.3 μl of the Reverse primer, two μl of the synthesized cDNA, and 7.4 μl of distilled water. The PCR temperature pattern for the respective genes was 95°C for 10 min for the first cycle, which continued with 45 cycles in two 95°C stages for 20 seconds and 60°C for 60 seconds. To analyze the data, first, the ΔCt of the gene in each sample was calculated as the difference between Ct of the corresponding gene and GAPDH gene as a reference gene ^16^. Reactions were performed in triplicate, and expression levels were calculated using the CT comparative method (2−ΔΔ*C*T) ^5^.

**Table 2:** Gene sequencing primer design used

**Genes **	**Primer sequence**
P53	F: TTTTCACCCCACCCTTCCCC
	R: CCTCAGACACACAGGTGGCA
Cytochrome C	F: TCCTCTACCCTCTATGCCAGGA
	R: AGCTGGGAACCATCATGTGC
GAPDH gene	F: GGAAAGCCTGCCGGTGACTA
	R: CACCCGGAGGAGAAATCGGG

###  Statistical analysis 

 To confirm data normality, homogeneity of variance, inter and intra-group differences for body weight, myocardium weight, and heart-to-body weight ratio, also the expression of P53 and cytochrome C genes (Shapiro-Wilk’s test, box plot, independent t-test, two-way analysis of variance, and Tukey’s post hoc test) were used respectively. Significance levels were set at *P* ≤ 0.05. All statistical calculations were performed using SPSS software version 25.

## Results

###  Anthropometric variables

 A total of 35 rats were included; two from Ex, one from Supp, and two from Con died during the study (Figure 1; Consolidated Standards of Reporting Trials (CONSORT) diagram). Physiologic characteristics and magnitude of change for all groups are provided in Table 3. We found significant effect for body mass after intervention in Ex (F1,28 = 15.8 *P* = 0.001), Supp (F1,28 = 11.7 *P* = 0.002) and Ex + Supp groups (F1,28 = 44.9 *P* = 0.01). In addition, the post hoc Tukey test showed a significant difference between the Con2 group and Ex, Supp, and Ex + Supp groups (*P* < 0.05), and the mean weight of rats in the Co2 group was significantly higher than the other three groups. However, no significant difference was observed in the body weights among the other three groups (*P* ˃ 0.05). Supp (F1,28 = 4.29 *P* = 0.049), Ex (F1,28 = 10.8 *P* = 0.003) and Ex + Supp groups (F1,28 = 013.4 *P* = 0.001) experienced remarkable changes in myocardium weight. Also, myocardium weight in the Con2 group was significantly lower than in other groups. (*P* < 0.05). However, no significant difference was observed in myocardium weight between groups (*P* ˃ 0.05). Furthermore, the effect of rosehip extract supplementation + endurance exercise (F1,28 = 12.19 *P* = 0.002), endurance exercise (F1,28 = 13.01 *P* = 0.001) and only rosehip extract supplementation (F1,28 = 5.56 *P* = 0.027) were significant on heart-to-body weight ratio. According to the results, post hoc demonstrated that level of heart-to-body weight ratio was significantly lower in the Con2 group than in the other three groups. (*P* < 0.05), however, no significant difference was observed between other groups (*P* ˃ 0.05). To evaluate the effect of aging on the desired indices, two control groups were compared using independent t-test. The results showed that the Con2 group’s body weight and heart weight were significantly higher than the con group (*P* > 0.01), but the ratio of heart weight to the body mass of the Con group was significantly larger than the Con group(*P* > 0.01).

**Table 3:** Physiologic characterstics and gene expression in male rats

**Group**	**Body weight(g)**	**Myocardium weight(g)**	**Myocardium weight/body weight(g/kg)**
Con	200 ± 20	0.22 ± 0.06	1.1 ± 0.13
Con2	267.4 ± 28.9^#^	0.43 ± 0.1^#^	1.61 ± 0.41^#^
Ex	171.8 ± 21.9^*^	0.59 ± 0.08^*^	3.48 ± 0.48^*^
Ex + Supp	176.71 ± 24.4^*^	0.63 ± 0.07^*^	3.56 ± 0.57^*^
Supp	210.1 ± 18.1^*^	0.54 ± 0.12^*^	2.57 ± 0.37^*^

Data are presented as mean ± SD; Con, three-month primary control; Con2, six-month control; Ex, endurance exercise; Ex + Supp, endurance exercise + rosehip supplement; Supp, rosehip supplement. ^*^significant differences with Con2 at *P* < 0.05; ^#^intergroup significant differences with the control group at *P* < 0.05.

###  Gene expression

**Figure 2:**
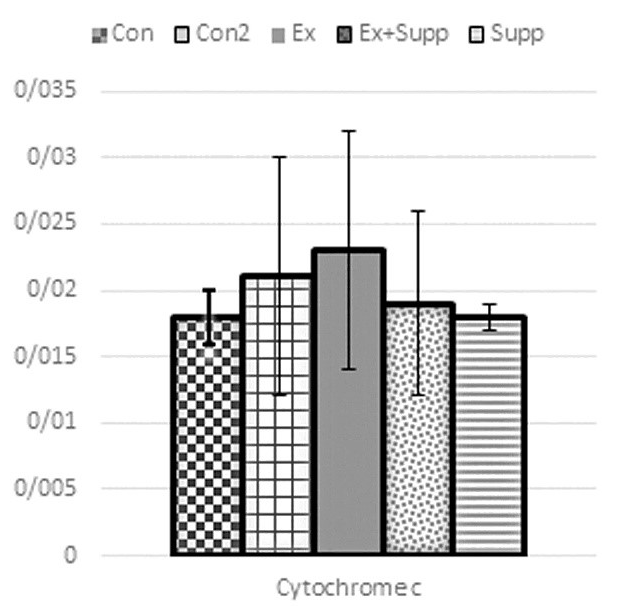
Cytochrome C gene expression.

**Figure 3:**
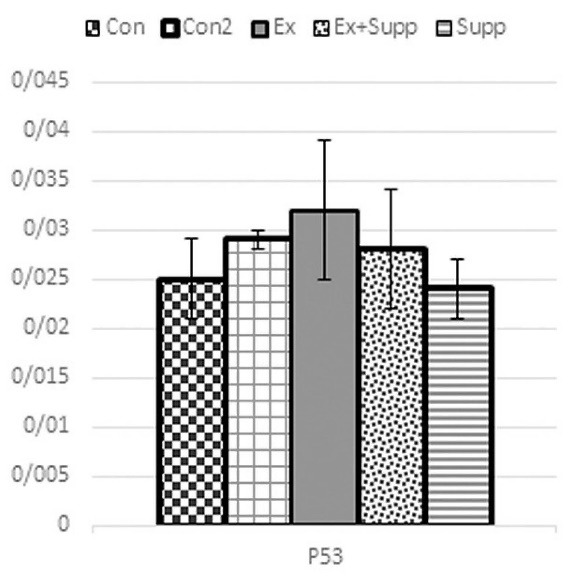
P53 gene expression.

 As you can see from Figures 2 and 3, following the intervention, Ex (P53: F_1,28_ = 1.98 *P* = 0.214; cytochrome C: F_1,28_ = 0.89 *P* = 0.534), Supp (P53: F_1,28_ = 0.046 *P* = 0.679; cytochrome C: F_1,28_ = 1.35 *P* = 0.169), and Ex + Supp groups (P53: F_1,28_ = 1.26 *P* = 0.431; cytochrome C: F_1,28_ = 1.14 *P* = 0.212) did not experienced significant changes in P53 and cytochrome C genes expression compared to Con2 group. There was no significant difference in cytochrome C (*P***=**0.09) and P53 (*P***=**0.32) gene expression between the Con and Con2 groups.

 Con, three-month basic control; Con2, six-month control; Ex, endurance exercising; Ex + Supp, endurance exercise + rosehip supplement; Supp, rosehip supplement.

## Discussion

 In this study we investigated the effect of endurance exercise accompanied by rosehip supplementation on apoptotic factors P53 and cytochrome C in the heart muscle of male rats. The results of this study show that exercise and Rosehip extract supplementation separately, and combined had no significant impact on cytochrome C and P53 gene expression.

 This is the first study that evaluates the effect of rosehip supplementation alone and togther with exercise on apoptosis-related markers. Previous studies have shown that exercise has protective effects against apoptosis by various mechanisms, such as direct changes in the expression of apoptotic genes, decreasing the release of mitochondrial apoptotic agents, changes in ROS production, and antioxidant system.^24-27^ Most studies have shown that cytochrome C levels are reduced by exercise.^26,28-30^ On the other hand, Ho et al showed that the expression of cytochrome C and caspase 3 in the heart muscle of the exercise group were significantly higher than the control group.^31^ Results of another study by Koff et al also showed that six months of endurance exercise accelerates apoptosis process in rats’ myocardium^10^. However, results of the present study revealed no significant alteration on cytochrome C gene expression after three months of endurance exercise.

 Moreover, previous studies showed that the changes in P53 levels due to exercise are contradictory. In some studies, the level of P53 expression is increased,^32,33^ but others reported a decreased in P53 after exercise.^34,35^ However, in the present study, the level of P53 remained unchanged compared to pre-exercise leves, which is in line with the obervations of Alipour et al^36^ and Sadeghi- Tabas et al.^37^ One of the reasons that cytochrome C and P53 expression remained unchanged in the present study was that the subjects in this study were healthy young rats. However, previous studies used old, ovariectomized, or diabetic rats, and the rate of apoptosis and cell death in these subjects are higher due to the oxidative stress and inflammatory factors, compared to the healthy young subjects. Moreover, compared to other studies that experienced significant changes in these factors, the intensity and duration of the exercise in our study were lower. The differences in study design may contribute to the observed contradicting results.

 Rosehip is another intervention that has gained attention as a possible anticarcinogenic plant.^21,38^ Previous studies have confirmed that this plant has cardioprotective, antiobesity, anti-inflammatory, antioxidative, antiaging, and neuroprotective properties.^21^ Studies showed that increasing oxidative stress is one of the reasons for the increased expression of P53 and cytochrome C releasing.^13^ In response to oxidative stress and increased P53 protein, Bax protein’s displacement and deposition in the outer mitochondrial membrane increases due to cytosolic JNK (c-Jun-N-terminal kinase) activation. This path inhibits Bcl-2 protein and interferes with the opening of mtPTP (Mitochondrial permeability transition pore), causing the pro-apoptotic agents of AIF and cytochrome C to be released into the cytosol. This process directly causes DNA fragmentation or through the Caspase cascade containing caspase-9 and caspase-3.^28,39-41^ So it seems that rosehip supplementation and exercise may lower the expression of P53 and cytochrome C by reducing stress and increasing antioxidant capacity.^21,34^ However, this reduction was not significant in our study, probably related to the frequency of administered doses of rose extract, age and health status of rats used in this study.^2^

 The present study was the first to evaluate the effect of rosehip and exercise alone or combined, on P53 and cytochrome C expression in cardiac muscle. Measuring morphological changes in the cardiac tissue, evaluating the expression of other proteins involved in the mitochondrial and external pathways of apoptosis, and measuring the protein content by Western blotting can provide additional information for better understanding the mechanisms involved in the observed outcome. Therefore, a definite statement on the effect of indicators related to myocardial apoptosis due to exercise and consumption of roses depends on further research and studies in this field.

## Conclusion

 Endurance exercise with rosehip supplementation probably modulates genes involved in apoptosis’s mitochondrial pathway, such as cytochrome C and P53. However, results of the present study revealed no significant impact of exercise or rosehip extract on these proteins. Duration and intensity of the exercise, and the ferequescy and dosage of the rosehip extract ued in this study may be responsible for these observations. Nethertheless, a definite statement on myocardial apoptosis and the concurrent effect of exercise and rose requires further investigation.

## Acknowledgments

 The authors would like to acknowledge the people who helped in this study.

## Author Contributions

**Conceptualization:** Mitra Abdollahi-Diba and Jabar Bashiri conceived the study.

**Methodology:** All Authors conducted the experiments.

**Validation:** Mitra Abdollahi-Diba and Jabar Bashiri evaluated the study validation.

**Formal Analysis:** Vahid Fekri-Kourabbaslou and Hadi Pourmanaf interpreted the data for the study.

**Investigation:** Mitra Abdollahi-Diba and Vahid Fekri-Kourabbaslou investigated previous literature.

**Resources:** All authors contributed substantially to the design of the work, drafted it, or revised it critically for important intellectual content.

**Data Curation:** Vahid Fekri-Kourabbaslou and Hadi Pourmanaf interpreted the data for the study.

**Writing—Original Draft Preparation:** Vahid Fekri-Kourabbaslou and Hadi Pourmanaf wrote the study’s first draft and final version.

**Writing—Review and Editing:** Vahid Fekri-Kourabbaslou and Hadi Pourmanaf answered the revision

**Visualization:** Mitra Abdollahi-Diba and Jabar Bashiri conceived the study

**Supervision:** This study conducted under the supervision of Jabar Bashiri.

**Project Administration:** Mitra Abdollahi-Diba was project administration.

**Funding Acquisition:** This research received no specific grant from any funding agency.

## Funding

 This research received no specific grant from any funding agency.

## Ethical Approval

 All protocol designs and surgical procedures were performed in compliance with the Guide for Using Animal Subjects. The Research Ethics Committee of Animal Experimentation of Tabriz Azad University, Iran approved the study (Ethical code: IR.IAU.TABRIZ.REC.124).

## Competing Interests

 The authors declare no conflict of interest and no competing interests.
